# Diffusion-Weighted Magnetic Resonance Diagnosis of Local Recurrences of Prostate Cancer after Radical Prostatectomy: Preliminary Evaluation on Twenty-Seven Cases

**DOI:** 10.1155/2014/780816

**Published:** 2014-02-16

**Authors:** Salvatore Francesco Carbone, Luigi Pirtoli, Veronica Ricci, Tommaso Carfagno, Paolo Tini, Augusto La Penna, Eleonora Cacchiarelli, Luca Volterrani

**Affiliations:** ^1^Istituto Toscano Tumori, via Taddeo Alderotti 26/N, 50139 Florence, Italy; ^2^Unit of Diagnostic Imaging, University Hospital of Siena, Policlinico S. Maria alle Scotte, Viale Bracci, 53100 Siena, Italy; ^3^Unit of Radiotherapy, University Hospital of Siena, Policlinico S. Maria alle Scotte, Viale Bracci, 53100 Siena, Italy

## Abstract

*Objectives.* To assess the diagnostic performance of diffusion-weighted MR imaging (DWI) in patients affected by prostatic fossa (PF) relapse after radical prostatectomy (RP) for prostatic carcinoma (PC). *Methods.* Twenty-seven patients showing a nodular lesion in the PF at T2-weighted MR imaging after RP, with diagnosis of PC relapse established by biopsy or PSA determinations, were investigated by DWI. Two readers evaluated the DWI results in consensus and the apparent diffusion coefficient (ADC) of the nodules, separately; a mean value was obtained (ADCm). *Results.* Relapses did not significantly differ in size in respect of postsurgical benign nodules. The DWI qualitative evaluation showed sensitivity, specificity, accuracy, ppv, and npv values, respectively, of 83.3%, 88.9%, 85.2%, 93.7%, and 72.7% (100%, 87.5%, 95.6%, 93.7%, and 100%, for nodules >6 mm). The intraclass correlation coefficient (ICC) for ADC evaluation between the two readers was 0.852 (95% CI 0.661–0.935; *P* = 0.0001). The ADCm values for relapses and benign nodules were, respectively, 0.98 ± 0.21 × 10^−3^ mm^2^/sec and 1.24 ± 0.32 × 10^−3^ mm^2^/sec (*P* = 0.006). Sensitivity, specificity, accuracy, ppv and npv of ADCm were, respectively, 77.8%, 88.9%, 81.8%, 93.3%, and 66.7% (93.3%, 87.5%, 85.4%, 93.3%, and 87.5% for nodules >6 mm). *Conclusions.* Diffusion-weighted MR imaging is a promising tool in the management of a hyperintense nodule detected by T2-weighted sequences. This might have a relevant importance in contouring radiotherapy treatment volumes.

## 1. Introduction

Prostate cancer (PC) is second in incidence among malignancies in men in Western countries [1]; the treatment of clinical localized disease is radical prostatectomy (RP) in many cases, and the decrease of the serum total prostate-specific antigen (PSAt) values below 0.1-0.2 ng/mL within 1 month after surgery documents complete tumour eradication [2]. However, a biochemical relapse occurs in 10%–53% of the patients undergoing RP within 5 years, defined as a PSAt increase in at least three consecutive samples [3]. This is usually the first sign of recurrence, often without any clinical or imaging evidence of a tumour mass; the clinical onset of recurrence follows after a median of 5 years [4]. At present, the pattern of relapse (prostatic fossa recurrence versus metastatic disease) is established on the basis of PSAt increase (PSA velocity or PSA doubling time) with slow increasing PSAt values (i.e., doubling time >12 months) suggesting local disease.

Early postoperative radiation therapy (RT) on PF may improve metastasis free and overall survival in cases at risk for LR [5]; however, in common practice, RT is performed in many cases at the occurrence of LR, with satisfactory results in terms of local control and prostate cancer-specific survival [6]. Radiotherapy as a salvage procedure is the treatment of choice and in such cases is usually performed on standardized volumes [7] corresponding to the PF. Presently, high doses (i.e., ≥70 Gy) are recommended and should be delivered to this whole clinical target volume [8]. However, the sophisticated RT techniques presently available, typically intensity modulated radiation therapy (IMRT), could allow such high doses to be delivered as a boost to the relapsing tumour mass, if detectable, and a lower dose (e.g., 60–64 Gy) to the “elective” volume, that is, the PF. This might result in decreasing RT side effects and complications. Thus, the detection of a mass in the PF and its reliable identification as a tumour relapse may be of the utmost importance.

Many imaging techniques have been investigated in order to individuate and characterize the local relapsing tumour mass after RP. Transrectal ultrasound (TRUS) has a poor capacity to differentiate post-surgical fibrous tissue from a relapse, but TRUS-guided biopsy is considered the gold standard of diagnosis of PC LR and should be attempted whenever possible. On the other hand, the European Association of Urology guidelines do not recommend biopsy for low level of PSA (<1 ng/mL) [9]. 18-Fluorocholine PET/CT scan is also reported to be useful, both as a follow-up procedure after RP in high-risk patients and in the case of a biochemical relapse (PSA > 1.4 ng/mL) [10].

Magnetic resonance imaging (MRI) is generally believed to be the most reliable diagnostic tool, when performed with functional imaging techniques in addition to conventional T2-weighted images. These techniques, such as dynamic contrast-enhanced MR imaging (DCE-MRI), proton spectroscopy (MRSI) [11–16], and diffusion-weighted MRI (DW-MRI, hereafter DWI), allow obtaining precious information about vascularization, metabolism, and tissue cellularity. In particular DWI, already recommended in the protocol for primary PC detection and staging [17], has recently been evaluated in identifying relapsing tumour in the PF after RP [18, 19]. In the present paper, we systematically evaluated a series of postoperative MRI examinations after RP and investigated the role of DWI in nodular lesions occurring in the PF, with the purpose of establishing the reliability of the method in diagnosis and localization of PF PC recurrences.

## 2. Methods and Materials

### 2.1. Patients

One hundred and fifty-two patients (pts) (mean age 68.2 y +/−  7.1) with a pathological staging after RP demonstrating T3a-b PCs were submitted to PSAt and MRI follow-up controls from January, 2008, to March, 2011 (i.e., from 3 to 41 months after surgery) at our institution. In 69 patients the conventional T2-weighted imaging demonstrated nodular findings in the PF: this subpopulation was the object of the present study. A PSAt value ≥0.2 ng/mL in three or more consecutive determinations was detected in 42 of them (“positive” cases), and an almost steady value <0.2 ng/mL in three determinations over a period of at least 18 months was observed in the other 27 ones (“negative” cases). Out of these 69 pts, only 29 entered the study: the remaining 40 were excluded due to presence of metastatic disease at nuclide bone scan, and/or CT scan, previous RT and/or hormone therapy and/or absence of DWI in the MR scan protocol. Finally, two positive cases were excluded from evaluation because of significant magnetic susceptibility artifacts (metallic clips in DWI images). The remaining 27 pts (18 cases with positive and 9 with negative PSAt determinations, as controls) are the subject of the present report. The standards of reference for positivity or negativity of the DWI results were similar to those outlined by the previous literature [13, 18]: TRUS-guided biopsy of the nodular lesions could confirm a relapsing PC in 7 pts, whereas in the other 11 ones a PSA decrease >50% was observed after RT. Out of the 83 pts with a negative T2-weighted imaging examination of the PF, 15 had positive PSAt determinations and were also submitted to RT on standard volumes. All the 27 patients included in the present study gave consent to the imaging investigations and to the anonymous use of clinical data.

### 2.2. Imaging

All MR examinations were obtained using a 1.5T MR scanner (Signa HDx Excite Twin Speed, GE Healthcare, USA). Images were acquired using commercially available balloon-covered expandable endorectal coils inflated with air (Endo ATD; Medrad, Pittsburgh, PA, USA) for signal reception, in combination with a four-channel phased array coil (Torso PA; GE Healthcare, USA) using a standardized protocol, as follows:

- T2-weighted fast spin echo (FSE) sequences on axial, sagittal, and oblique coronal planes, perpendicular and parallel to prostatic urethra (TR/TE 4600/105 msec, bandwidth 20.8 kHz, matrix 288 × 256, FOV (cm) 24 × 24, thickness/gap (mm) 4/0, and NSA-6);
- DWI spin echo (SE) and echo planar imaging (EPI) sequences, with 90° and 180° RF pulses, on axial plane (TR/TE 3675/119 msec, bandwidth 167 kHz, matrix 128 × 128, FOV (cm) 24 × 24, thickness/gap (mm) 4/0 mm, and NSA 4); to reduce magnetic susceptibility artifacts, a spectral fat-saturation pulse was added; diffusion motion probing gradients with *b*-values of 0 and 600 s/mm^2^ were applied on the three orthogonal directions *z*, *x*, and *y*, with vectorial imaging reconstruction (DW images).

**Figure 1:**
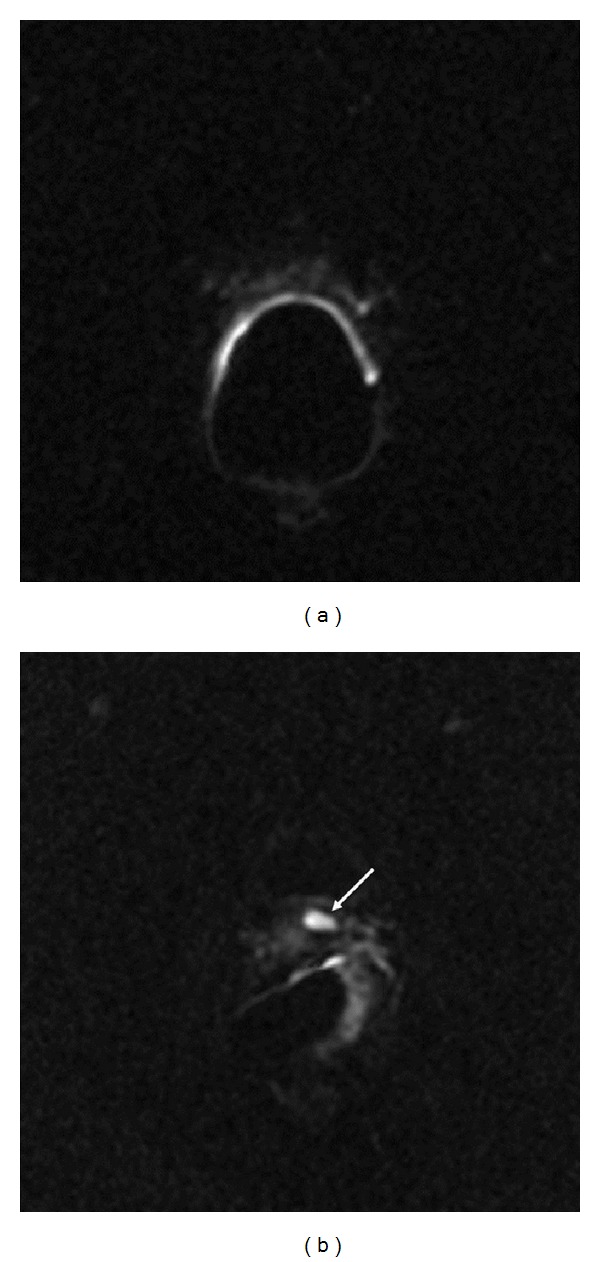
DWI qualitative scoring. The observers attributed score 0 to the absence of clear-cut hyperintense lesion in the PF area (a) and score 1 to a focal hyperintensity (arrow, (b)) attributable to a relapse.

Two abdominal radiologists, each unaware of the results of the PSA values and of any other imaging examination, reviewed the MR imaging on a commercial workstation (Advantage Windows, release 4.4, GE Healthcare, USA). A locoregional relapse was suspected whenever a hyperintense nodule (compared to signal of nearest obturator muscle) in the area of PF was observed on conventional T2-weighted images, in more than one plane. The size of the nodules was measured, as the maximum diameter observed in an axial plane, and recorded. The same nodules were reevaluated in DWI (*b* = 600 sec/mm^2^) assuming as a reference the corresponding T2-weighted images. The 1 and 0 score were assigned, respectively, for presence or absence of hyperintensity, compared to background, in the site of the lesion detected in T2-weighted images (Figure 1). The two readers made this evaluation in consensus. The same operators obtained a quantitative DWI assessment separately, at least one month later, by apparent diffusion coefficient (ADC) maps, calculated using a commercially available software (Functool release 4.4.05, Advantage Windows 4.4, GE Healthcare, USA). Each operator marked an oval-shaped region of interest (ROI) on pathologic area using as a reference DWI images (*b* = 0 mm/sec^2^) previously recognized as DWI score 1, taking into account as a reference the corresponding T2-weighted image. This ROI was automatically pasted on the ADC map, in order to obtain the ADC values of the lesion, each pixel in ADC maps resulting the application of the pixel-by-pixel Stejskal-Tanner monoexponential relationship ADC = Log(SI_0_ − SI_1_)/*b*_1_ − *b*_0_ [20], where SI_0_ and SI_1_ are, respectively, the signal intensity at *b*-value of *b*_0_ = 0 and *b*_1_ = 600 sec/mm^2^.

### 2.3. Statistical Analysis

We used nonparametric statistical tests (Mann-Whitney; Spearman's) due to the small number of pts included in the present reports. The intra-class correlation coefficient (ICC) [21] was used to evaluate the interobserver agreement for ADC results. The qualitative DWI data (the 0/1 scoring system, as above) and the mean ADC values (ADCm) resulting by the separate observations of the two readers were used for further diagnostic performance evaluations, as follows. Sensitivity, specificity, accuracy, positive, and negative predictive values (ppv and npv, resp.) were calculated by the receiver-operating characteristic curves (ROC). Commercially available software for statistical analysis was used for these purposes (SSPS 17.0, Chicago, USA).

## 3. Results

Biochemical relapses occurred in 57 out of the 152 pts considered in the preliminary evaluation (37.5%), consistently with the available data of post-RP series of pathological stage T3 PCs [3]. As regards the subject of this paper, PSAt value was 0.29 ± 0.22 ng/mL (95% CI 0.12–0.46) for benign lesions and 1.87 ± 1.29 ng/mL (95% CI 1.22–2.51) for relapses (*P* < 0.0001). The size of the lesions at the T2-weighted sequences did not differ significantly between relapses (11.98 ± 5.05 mm; 95% CI 9.46–14.49) and benign nodules (12.1 mm ± 5.36 mm; 95% CI 7.88–16.12) (*P* = 0.941) out of the 27 cases included in the study. Nodule size was larger than 6 mm in 23 patients. The size of the “malignant” nodules and the PSA values were not significantly correlated (rho: −0.081; *P* = 0.687).

**Figure 2:**
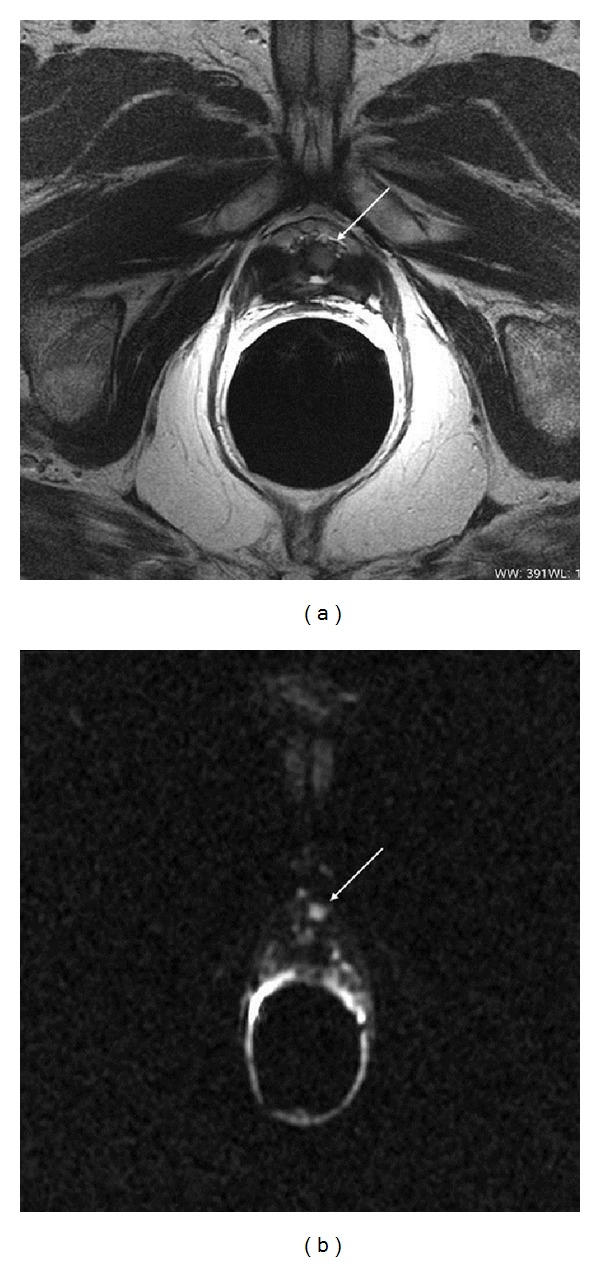
Relapse after radical prostatectomy. Arrows show a nodular intermediate signal intensity in the T2-weighted image (a), characterized by hyperintensity in DWI (score DWI = 1: (b)).

**Figure 3:**
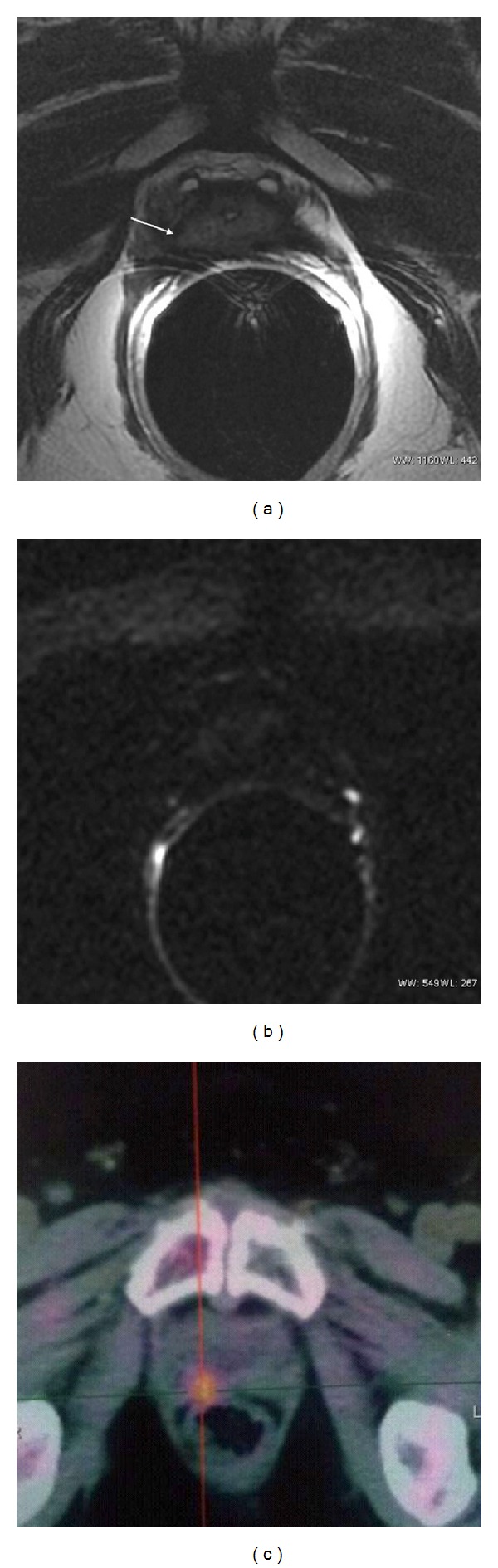
DWI, false negative. Patient with a glandular apical residual prostate tissue and a progressive increase of PSAt (last value before DWI: 0.61 ng/mL). The T2-weighted image (a) shows the minimal residual prostate tissue with a small bulging in the right paramedian site (arrow). No detectable hyper intensity was visualized in DW image (score DWI = 0, (b)); 18F-choline PET-CT (c) showed a focal uptake at the site of the described bulging. The post-RT decrease of PSAt under 0.2 ng/mL was a further standard of reference in this case.

**Figure 4:**
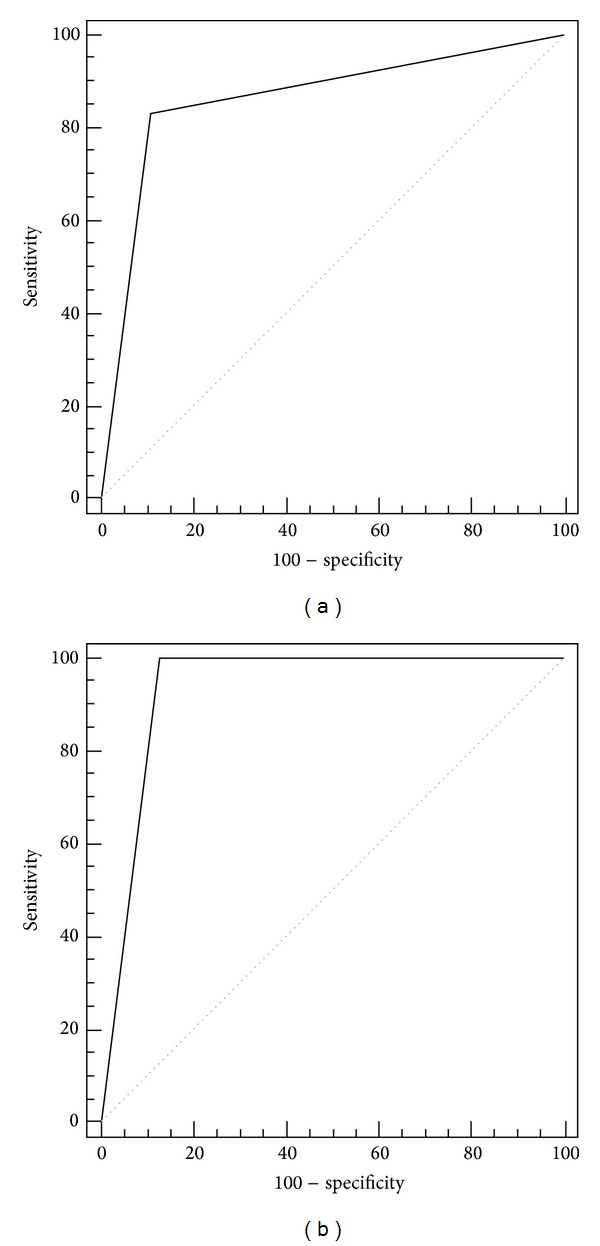
Receiver-operating characteristic curves of DWI score in the whole pts series (a) and in PF nodules with size >6 mm (b). The area under the curve increased for these last cases.

The DWI qualitative score analysis of the hyperintense nodules, carried out in consensus between the two observers, showed 15 true positives, 8 true negatives, 3 false negatives, and 1 false positive (Figure 2, Table 1). In all 3 false negatives the size of the lesion was <6 mm (Figure 3). Sensitivity, specificity, accuracy, ppv, and npv were, respectively, 83.3%, 88.9%, 85.2%, 93.7%, and 72.7%, out of the whole series. Out of the 23 patients showing nodules >6 mm, the reliability of the diagnostic parameters was improved: 100%, 87.5%, 95.6%, 93.7%, and 100%, respectively (Figure 4, Table 2).

**Figure 5:**
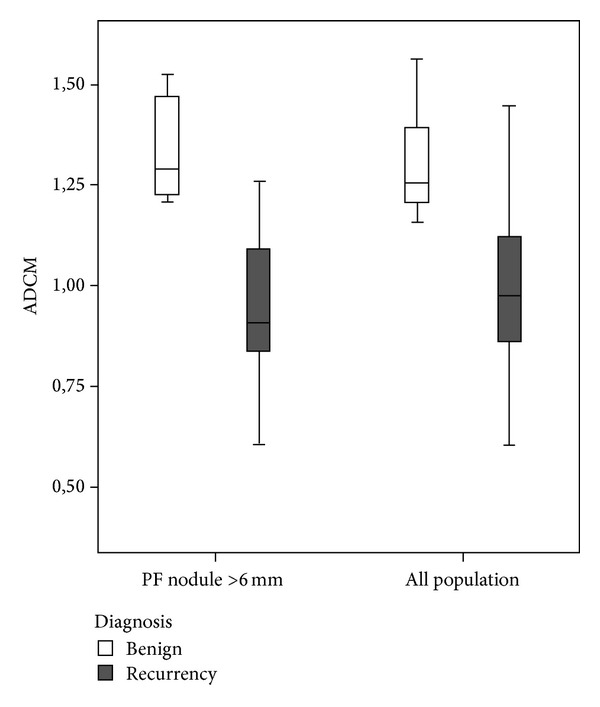
ADCm boxplots for nodules >6 mm and for the whole series. Note the lack of a significant overlapping between recurrences and benign nodules when nodules size <6 mm is excluded.

About the quantitative evaluation, the interobserver agreement showed an ICC of 0.852 (95% CI 0.661–0.935; *P* = 0.0001). The mean ADC values (ADCm) obtained from the observations of the two readers were 0.98 ± 0.21 × 10^−3^ mm^2^/sec (95% CI 0.88–1.08) for the relapses and 1.24 ± 0.32 × 10^−3^ mm^2^/sec (95% CI 0.99–1.09) for the benign nodules (*P* = 0.006), with a cut-off value of 1.13 × 10^−3^ mm^2^/sec (Figure 5). Sensitivity, specificity, accuracy, ppv, and npv of ADCm were, respectively, 77.8%, 88.9%, 81.8%, 93.3%, and 66.7%. Also for ADCm values, as for the above DWI qualitative analysis, the diagnostic parameters showed an improved reliability in nodules >6 mm (93.3%, 87.5%, 85.4%, 93.3%, and 87.5%, resp.) (Tables 1 and 2).

## 4. Discussion

DWI provides reliable data concerning tissue cellularity; cell membranes, in fact, limit water proton diffusion [20]. In tumour tissues, such as PC, the hypercellularity is a constraint to proton mobility, differently from other tissues where water molecules move freely in a wide extracellular space [22]. This translates in a natural contrast-based imaging differentiation between tumour and other lesions (e.g., inflammatory or fibrotic), from a qualitative point of view. Also quantitative determinations can be achieved, with ADC values that are significantly lower in tumour than in other lesions and in normal tissue. On these bases, DWI received a considerable interest in the literature concerning primary diagnosis of PC [23–27]. Moreover, DWI has recently been considered as a useful tool in evaluation of PF in patients with biochemical relapse of PSA after primary therapy: ESUR guidelines on prostate MR recommend its use in addition to T2-weighted imaging and DCE in these cases [17].

In our experience, the qualitative DWI examinations achieved 15 true positives, 8 true negatives, 3 false negatives, and 1 false positive, with sensitivity, specificity, accuracy, ppv, and npv values, respectively of 83.3%, 88.9%, 85.2%, 93.7%, and 72.7%, out of the whole series. The three false-negative lesions had a size <6 mm; this is probably due to a low in-plane spatial resolution, as well as to a low signal-to-noise ratio in the DW images; this drawback could be overcome by a 3T MR system [18]. The only false positive could be due to the presence of urine in the urethral anastomosis, close to the lesion, leading to a shine-through artifact. The reliability of the diagnostic parameters was improved in the 23 pts showing nodules >6 mm and 100%, 87.5%, 95.6%, 93.7%, and 100% values were obtained, respectively.

The quantitative ADCm evaluation showed sensitivity, specificity, accuracy, ppv, and npv values that were, respectively, 77.8%, 88.9%, 81.8%, 93.3%, and 66.7%. Also for ADCm values, as for DWI qualitative analysis, the diagnostic parameters showed an improved reliability in nodules >6 mm (93.3%, 87.5%, 85.4%, 93.3%, and 87.5%, resp.). The ADCm values here obtained for LR (cut-off of 1.13 × 10^−3^ mm^2^/sec) are slightly higher than those reported by other authors [19] that used higher *b*-values for DWI. This could be explained by the decrease of pseudodiffusion, due to perfusion, that occurs by increasing *b*-values and that in turn results in reduced ADC values. However, it should be pointed out that a significant suppression of the perfusion component is generally observed even with *b*-values of 300–400 s/mm^2^ [28] and that the so-called slow-component perfusion largely predominates for higher *b*-values, due to the intravoxel incoherent motion (IVIM). This, consequently, results in a further small decrease in ADC by increasing *b*-values over those adopted in the present study, high enough to provide an acceptable signal-to-noise ratio in the DW images obtained with the 1.5T MR system that we used. In fact, the choice of a *b*-value of 600 s/mm^2^ can be considered low for ADC calculation, as suggested by ESUR guidelines [17]; This can explain the worst diagnostic performance of our quantitative evaluation, if compared to the DWI score that we used for qualitative one. Further acquisitions with higher *b*-values could increase the diagnostic performance. Nevertheless, it should be considered that our first goal was to assess the reliability of a fast qualitative evaluation of the PF in term of presence/absence of relapse; in this attempt an adequate signal-to-noise ratio is ensured by not exceedingly high *b*-value at 1.5T. Another technical drawbacks are magnetic susceptibility artifacts, due to metallic clips or to the air-filled balloon, in the interface with the rectum, that could lead to an unreliable evaluation of some lesions, especially in posterior portion of the PF (false positive or unsatisfactory images). Finally, a limitation of the present analysis is also the small number of patients. This aspect and the lack of significant correlations between the PSAt serum levels and the size of the relapse deserve further consideration, in the attempt to identify a useful PSAt threshold value for the reliability of DWI and of other functional MRI techniques.

Despite these limitations, our results are encouraging and they are comparable to other data regarding DWI in detecting local recurrences, obtained also with higher field MR system [18, 29]. Particularly, a single-center study documented no significant differences of 1.5T versus 3.0T in detection of local recurrences [29]; in this work the median tumour size was of 0.26 cm^3^, with a significant cut-off PSAt of 0.3 ng/mL. In another single-center prospective study, authors report combined T2-weighted and DWI sensitivity 93–98%, specificity 89–96%, and accuracy 86–92%, depending on size of the lesion and *b*-value, using a 3T MR system [18]. This last MR equipment could solve problems related to low spatial resolution of DWI sequences and could give the possibility to use higher *b*-values, as the reported diagnostic performance seems to increase with higher *b*-values (*b* = 3000 s/mm^2^) also for small lesions (4–8 mm) [18]. However, 3T MR facilities are less in current use than 1.5T systems; if our data will be confirmed by more large population studies, the translation of this technique in common clinical practice could be more feasible. DWI also shows diagnostic results comparable with other functional techniques: DCE-MR and MRSI show sensitivity, specificity, accuracy, ppv, and npv values, respectively, in the range of 84%–95%, 75%–100%, 86%–94%, 92%–100%, and 57%–88%, that are generally higher than those achieved by T2-weighted MR imaging and also by PET-CT investigations, at least for LR with mean diameters in the range from 6 mm to 15 mm [30]. Particularly, DCE-MRI seems to be more accurate if achieved with a 3T MR system [18]. On the other hand, DCE-MRI requires contrast medium administration, which sometimes could be not tolerated, and MRSI needs long acquisition and postprocessing times, and a long training period for the operator. On the contrary, DWI acquisition is achieved in 2-3 minutes and the ADC maps are automatically obtained by the workstation.

Compared with MR imaging, PET-CT scan deserves a particular mention due to the capability to differentiate local from systemic relapses. In particular, the use of choline as tracer is considered a reliable PCa biomarker for its role in cellular membrane metabolism. In a recent review, Cho-PET-CT was reported in patients with biochemical recurrence after RP or RT, with sensitivity ranging from 38% to 98%, depending on different issues like PSA value and type of treatment [10]. However, in patients treated with RP, Cho-PET-CT detection rate is too low for PSA value <1 ng/mL; at this value, salvage RT achieves suboptimal results in terms of outcome [9]. Moreover, in patients with PSA > 2 ng/mL and negative imaging, Cho-PET-CT scan is positive only in 28% cases [9]. At least, PET-CT scan is not widely available.

Of course, the present report needs confirmation on the grounds of a larger series of patients.

## 5. Conclusions

**Figure 6:**
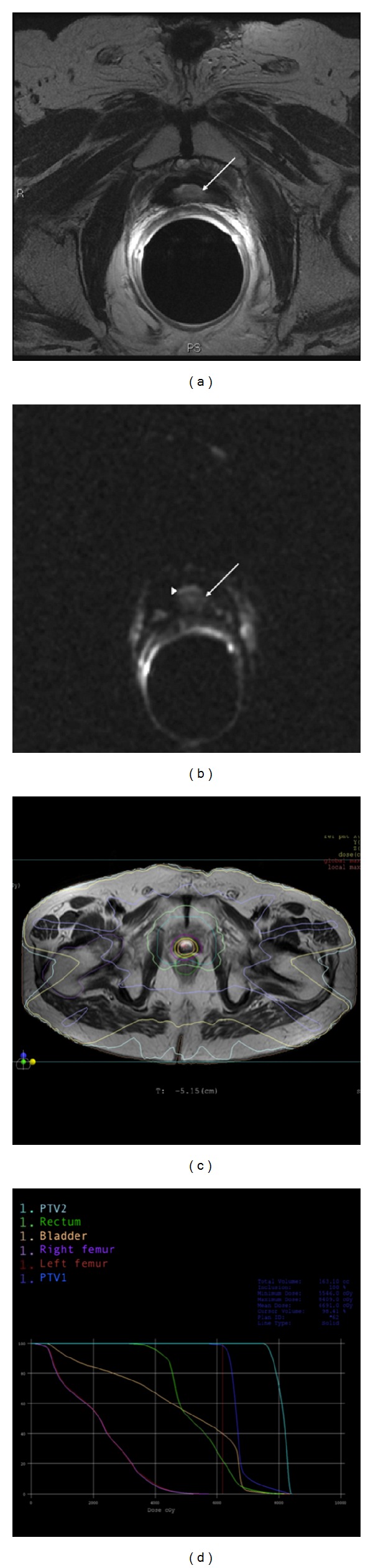
RT simulation plan: a paraurethral nodule suspected for relapse (white arrow) is shown by T2 images in PSA-positive patients (a); DWI score 1 qualitative evaluation (white arrow, suspected nodule; arrowhead, shine-through artifact of urine) (b); IMRT simultaneous boost (SIB) treatment plan (PTV1: 64 Gy delivered to the PF; PTV2: 74 Gy delivered to the nodule) (c); dose-volume histograms of the IMRT SIB plan (d).

Presently, the DWI characterization of a nodule in PF, detected by a previous T2-weighted MR imaging in patients showing a PSAt increase after RP for PC, can be considered appropriate as an alternative to more sophisticated MRI techniques, not widely available. Further studies are mandatory to confirm the preliminary results reported here. The reliable identification and localization of the relapse by MR imaging may be of the utmost importance mainly in RT salvage treatment (i.e., a very frequent occurrence), a useful and practical tool for targeting it with a boost dosage in the course of IMRT (Figure 6), or of other advanced irradiation techniques.

## Figures and Tables

**Table 1:** Qualitative and quantitative imaging assessment in respect of the standards of reference.

	DWI (size > 6 mm)		ADC* (size > 6 mm°)	
Standard of reference	No relapse	Relapse	No relapse	Relapse
No relapse	8 (7)	1 (1)	8 (7)	1 (1)
Relapse	3 (0)	15 (15)	4 (1)	14 (14)

Data regarding PF nodules >6 mm are shown in brackets; *cut-off 1.13 × 10^−3^ mm^2^/sec and °cut-off 1.2 × 10^−3^ mm^2^/sec.

**Table 2:** Performance (%) of DWI; data regarding PF nodules >6 mm are shown in brackets.

	Sensitivity	Specificity	Accuracy	ppv	npv	Cut-off
DWI score	83.3 (100)	88.9 (87.5)	85.2 (95.6)	93.7 (93.7)	72.7 (100)	
ADCm	77.8 (93.3)	88.9 (87.5)	81.8 (85.4)	93.3 (93.3)	66.7 (87.5)	1.13 (1.2)
